# Transmission dynamics for Methicilin-resistant Staphalococous areus with injection drug user

**DOI:** 10.1186/s12879-018-2973-4

**Published:** 2018-02-07

**Authors:** Rebekah Wagner, Folashade B. Agusto

**Affiliations:** Department of Ecology and Evolutionary Biology, University of Kansas, Lawrence, 66045 KS USA

**Keywords:** Methicillin-resistant, Injection drug users, Sensitivity analysis, Risk factors, Control strategies, 92B05, 93A30, 93C15

## Abstract

**Background:**

Methicillin-resistant Staphylococcus aureus (MRSA) is a bacterial pathogen resistance to antibiotics including methicillin. The resistance first emerged in 1960 in a healthcare setting only after two years of using methicillin as a viable treatment for methicillin-susceptible Staphylococcus aureus. MRSA leads to infections in different parts of the body including the skin, bloodstream, lungs, or the urinary tract.

**Methods:**

A deterministic model for methicillin-resistant Staphylococcus aureus (MRSA) with injection drug users is designed. The model incorporates transmission of MRSA among non-injection drug users and injection drug users (IDUs) who are both low-and high-risk users. A reduced MRSA transmission model with only non-IDUs is fitted to a 2008-2013 MRSA data from the Agency for Healthcare and Research and Quality (AHRQ). The parameter estimates obtained are projected onto the parameters for the low-and high-risk IDUs subgroups using risk factors obtained by constructing a risk assessment ethogram. Sensitivity analysis is carried out to determine parameters with the greatest impact on the reproduction number using the reduced non-IDUs model. Change in risk associated behaviors was studied using the full MRSA transmission model *via* the increase in risky behaviors and enrollment into rehabilitation programs or clean needle exchange programs. Three control effectiveness levels determined from the sensitivity analysis were used to study control of disease translation within the subgroups.

**Results:**

The sensitivity analysis indicates that the transmission probability and recovery rates within the subgroup have the highest impact on the reproduction number of the reduced non-IDU model. Change in risk associated behaviors from non-IDUs to low-and high-risk IDUs lead to more MRSA cases among the subgroups. However, when more IDUs enroll into rehabilitation programs or clean needle exchange programs, there was a reduction in the number of MRSA cases in the community. Furthermore, MRSA burden within the subgroups can effectively be curtailed in the community by implementing moderate- and high-effectiveness control strategies.

**Conclusions:**

MRSA burden can be curtailed among and within non-injection drug users and both low-and high-risk injection drug users by encouraging positive change in behaviors and by moderate- and high-effectiveness control strategies that effectively targets the transmission probability and recovery rates within the subgroups in the community.

## Background

Methicillin-resistant Staphylococcus aureus (MRSA) is a bacterial pathogen resistance to antibiotics including methicillin. It first emerged in 1960 in a healthcare setting only after two years of methicillin being used as a viable treatment for methicillin-susceptible Staphylococcus aureus. MRSA has long been isolated in the community since its emergence in the hospital setting.

Transmission of MRSA is achieved either by direct contact with a colonized patient or indirect contact. Indirect transmission occurs when the bacteria is transferred from an infected person to a fomite where it can stay infectious for months [1].

The epidemic of MRSA among injection drug users (IDUs) began in 1981 and had since become endemic in the community [2]. A number of research studies have studied MRSA among IDUs by tracking registered IDUs [3–6]. For instance, Fleisch et al. [5] followed 31 MRSA infected IDUs and found that 19 individuals developed secondary, life-threatening infections such as septic arthritis, endocarditis, pneumonia, and osteomyelitis. Another study by Binswanger et al. [4] found that out of a population of 169 IDUs, 29 individuals displayed subcutaneous inflammation and infection.

Injection drug users present a unique set of behavioral factors that all accumulate to an increased risk of MRSA transmission and infection. One of those risk factors is trauma to the skin which creates opportunities for MRSA bacteria to access their soft tissue during the act of injecting their drug of choice. The proceeding factors all vary depending on habits and behaviors associated with injection drug use that will be detailed in the “Parameter estimation” section.

Our goal was to understand the transmission dynamics of MRSA in a community with IDUs and to investigate the impact of drug rehabilitation programs, intervention, education, clean needle exchange programs, and so forth [7, 8] as part of control measures to curtail MRSA transmission in the community. Therefore, we formulate a deterministic model for MRSA transmission dynamics which include non-IDUs and two subgroups of IDUs with different risk associated behaviors (i.e., low- and high-risk behaviors).

This paper is organized as follows: in “Model formulation” section, we present the MRSA transmission model and calculate the basic reproduction number. In the “Parameter estimation” section, we estimate the values of the model parameters. In the “Sensitivity analysis” section, we carry out a sensitivity analysis to identify the model’s parameters with the most impact on our response function. Using the results obtained from the sensitivity analysis, we investigate in “Control measures” section the impact of some control strategies on MRSA transmission in the community.

## Method

### Model formulation

To formulate the methicillin-resistant Staphylococcus aureus transmission model with injection drug users, individuals in the community were divided into three subgroups, non-injection drug users (IDUs), low-risk IDUs, and high-risk IDUs in order to understand the interactions between these populations. Each subgroup was subsequently divided into three compartments according to their disease status. Thus, we have uncolonized susceptible individuals (*U*_*i*_), colonized individuals (*C*_*i*_), and infected individuals (*I*_*i*_), where *i*=*N*,*L*,*H* for non-IDUs, low-risk IDUs, and high-risk IDUs. These premises lead to the following total population

$$\begin{array}{@{}rcl@{}} N &=& U_{N} + C_{N} + I_{N} + U_{L} + C_{L} + I_{L} + U_{H} + C_{H} + I_{H} \end{array} $$

**Fig. 1:**
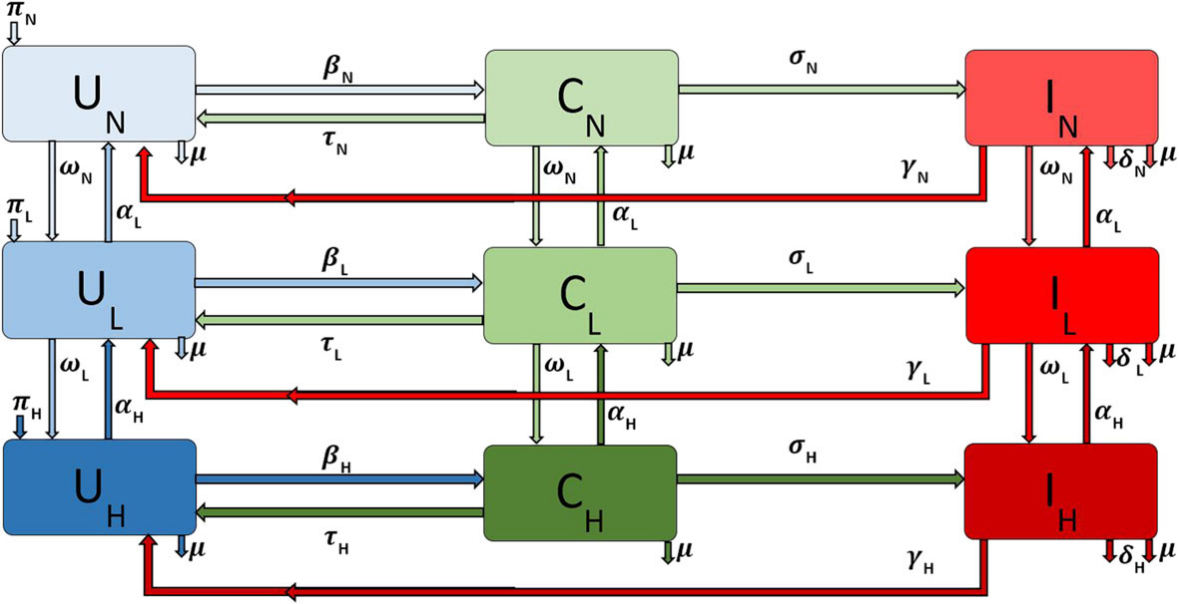
Flow diagram of the MRSA model (1) with IDUs

The MRSA transmission dynamics with IDUs is given by the following system of ordinary differential equations and depicted in Fig. 1. The associated variables and parameters are described in Table 1.

1 $$\begin{array}{@{}rcl@{}} \frac{dU_{N}}{dt} &=& \pi_{N} + \alpha_{L} U_{L} + \tau_{N} C_{N} + \gamma_{N} I_{N}  \\&&- \frac{\beta_{N} (C_{N}+I_{N}+C_{L}+I_{L}+C_{H}+I_{H})}{N} \\ && - (\omega_{N}+\mu) U_{N} \\ \frac{dC_{N}}{dt} &=& \alpha_{L} C_{L} + \frac{\beta_{N} (C_{N}+I_{N}+C_{L}+I_{L}+C_{H}+I_{H}))}{N}  \\&& - (\tau_{N}+\sigma_{N}+\omega_{N}+\mu) C_{N} \\ \frac{dI_{N}}{dt} &=& \alpha_{L} I_{L} + \sigma_{N} C_{N} - (\omega_{N} +\gamma_{N}+\mu+\delta_{N})I_{N} \\ \frac{dU_{L}}{dt} &=& \pi_{L}+ \omega_{N} U_{N} + \alpha_{L} U_{L} \\&&- \frac{\beta_{L} (C_{N}+I_{N}+C_{L}+I_{L}+C_{H}+I_{H})}{N} \\ && + \tau_{L} C_{L} + \gamma_{L} I_{L} - (\omega_{L}+\alpha_{L}+\mu)U_{L}  \\ \frac{dC_{L}}{dt} &=& \omega_{N} C_{N} + \alpha_{H} C_{H} + \frac{\beta_{L} (C_{N}+I_{N}+C_{L}+I_{L}+C_{H}+I_{H})}{N} \\ && - (\tau_{L}+\omega_{L}+\alpha_{L}+\sigma_{L}+\mu)C_{L} \\ \frac{dI_{L}}{dt} &=& \omega_{N} I_{N} + \alpha_{H} I_{H} + \sigma_{L} C_{L} - (\omega_{L}+\alpha_{L}+\gamma_{L}+\mu+\delta_{L})I_{L} \\ \frac{dU_{H}}{dt} &=& \pi_{H} + \omega_{L} U_{L} + \gamma_{H} I_{H} + \tau_{H} C_{H}  \\&&- \frac{\beta_{H} (C_{N}+I_{N}+C_{L}+I_{L}+C_{H}+I_{H})}{N} \\ && - (\alpha_{H}+\mu)U_{H} \\ \frac{dC_{H}}{dt} &=& \omega_{L} C_{L} + \frac{\beta_{H} (C_{N}+I_{N}+C_{L}+I_{L}+C_{H}+I_{H})}{N}  \\&&- (\tau_{H}+\alpha_{H}+\sigma_{H}+\mu)C_{H} \\ \frac{dI_{H}}{dt} &=& \omega_{L} I_{L} + \sigma_{H} C_{H} - (\alpha_{H}+\gamma_{H}+\mu+\delta_{H})I_{H}. \end{array} $$

**Table 1:** Variable and parameter descriptions for the MRSA model (1) per day

Variable	Description
*U*_*N*_,*U*_*L*_,*U*_*H*_	Population of uncolonized non-IDUs, low-risk IDUs, and high-risk IDUs
*C*_*N*_,*C*_*L*_,*C*_*H*_	Population of colonized non-IDUs, low-risk IDUs, and high-risk IDUs
*I*_*N*_,*I*_*L*_,*I*_*H*_	Population of infected non-IDUs, low-risk IDUs, and high-risk IDUs
Parameter	Description
*π*_*N*_, *π*_*L*_, *π*_*H*_	Natural birth rate
*β*_*N*_,*β*_*L*_,*β*_*H*_	Transmission probability per contact with colonized or infected population
*σ*_*N*_,*σ*_*L*_,*σ*_*H*_	Progression rate of colonized population
*γ*_*N*_,*γ*_*L*_,*γ*_*H*_	Recovery rate of infected population
*τ*_*N*_, *τ*_*L*_, *τ*_*H*_	Decolonization rate of colonized population
*ω*_*N*_,*ω*_*L*_,*ω*_*H*_	Increased IDU risk behavior
*α*_*N*_,*α*_*L*_,*α*_*H*_	Decreased IDU risk behavior
*μ*	Natural death rate
*δ*_*N*_,*δ*_*L*_,*δ*_*H*_	Death rate due to disease

The parameter *π*_*N*_ is the recruitment rate into the uncolonized non-injection drug users (*U*_*N*_) subgroup. The parameter *μ* is the natural death rate in each sub-population. The force of infection of the non-injection drug users is given by

$$\frac{\beta_{N} (C_{N}+I_{N}+C_{L}+I_{L}+C_{H}+I_{H})}{N}, $$

where *β*_*N*_ is the probability of an uncolonized individual becoming colonized upon being exposed to MRSA bacteria through contact with colonized or infected individuals in either the non-injection drug users class or the low-risk class or the high-risk classes. Once individuals in the colonized class are decolonized at the rate *τ*_*N*_ [9], they move back to the uncolonized class. However, as the disease progresses, the colonized non-IDUs (*C*_*N*_) move to the infected class at rate *σ*_*N*_. They recover at the rate *γ*_*N*_ and thus move from the infected compartment (*I*_*N*_) back to the uncolonized class (*U*_*N*_). The parameters and transitions for the low-risk and high-risk populations are similarly defined (with the subscript *N* replaced by *L* and *H* respectively).

If non-IDUs engage in risky behavior resulting from injection drug use, we assume that this behavior initially involves a less risky use of drugs and as such these individuals leave the non-IDUs subgroup at rate *ω*_*N*_ into the low-risk IDU subgroup. However, if these individuals decrease their risky behaviors either by enrolling in drug rehabilitation programs [3, 10] or by other kinds of interventions they move back into the non-IDU subgroup at the rate *α*_*L*_.

Individuals in the low-risk subgroup who further engage in increased risky behaviors move into a high-risk injection drug users subgroup at the rate *ω*_*L*_. As with the low-risk individuals, these individuals may stop injecting drugs when they enroll in a drug rehabilitation or needle exchange programs [3, 10]. We assume these processes are not instantaneous. Hence they first enter the low-risk subgroup at rate *α*_*H*_. Injection drug users harbor more *S. aureus* bacteria compare to non users [11], and required prolonged treatment [12, 13], thus we assume that these risky behaviors increases the rates of MRSA transmission [14, 15].

### The basic reproduction number

The basic reproduction number ($\mathcal {R}_{0}$) of the MRSA model (1) with IDUs is given below; the theoretical study of the model basic properties is stated in Appendix A under ‘Analysis of the model’ and the calculations of $\mathcal {R}_{0}$ are given in Appendix B.

2 $$\begin{array}{@{}rcl@{}} \mathcal{R}_{0} &=& \mathcal{R}_{N} + \mathcal{R}_{L} + \mathcal{R}_{H}, \end{array} $$

where

$$\begin{aligned} \mathcal{R}_{N} &= \beta_{N}U^{*}_{N}\left[k_{7}\omega_{N}(k_{3}k_{8}+\omega_{L}k_{3}+k_{8}\alpha_{L})\sigma_{L} \right.\\ &\quad +(k_{6}k_{8}\,+\,\omega_{N}k_{8}+\omega_{N}\omega_{L}-\omega_{L}\alpha_{H})(k_{5}k_{7}-\omega_{L}\alpha_{H})\sigma_{N} \\ &\quad +\omega_{N}\omega_{L}(k_{3}k_{6}+k_{3}\alpha_{H}+\alpha_{H}\alpha_{L}-\omega_{N}\alpha_{L})\sigma_{H} \\ &\quad \left.+(k_{5}k_{7}+k_{7}\omega_{N}+\omega_{L}\omega_{N}-\omega_{L}\alpha_{H})(k_{3}k_{6}k_{8}\right. \\& \quad \left.-k_{3}\omega_{L}\alpha_{H}-k_{8}\omega_{N}\alpha_{L})\right] /N^{*}(k_{3}k_{6}k_{8}-k_{3}\omega_{L}\alpha_{H}\\&\quad-k_{8}\omega_{N}\alpha_{L})(k_{2}k_{5}k_{7} -k_{2}\omega_{L}\alpha_{H}-k_{7}\omega_{N}\alpha_{L})\\ \mathcal{R}_{L} &= \beta_{L}U^{*}_{L}\left[k_{2}k_{7}(k_{3}k_{8}+\omega_{L}k_{3}+\alpha_{L}k_{8})\sigma_{L} \right.\\ &\quad +k_{7}\alpha_{L}(k_{6}k_{8}+k_{8}\omega_{N}+\omega_{N}\omega_{L}-\omega_{L}\alpha_{H})\sigma_{N} \\ &\quad +k_{2}\omega_{L}(k_{3}k_{6}+k_{3}\alpha_{H}+\alpha_{L}\alpha_{H}-\omega_{N}\alpha_{L})\sigma_{H} \\ &\quad \left.+(k_{2}k_{7}+k_{2}\omega_{L}+k_{7}\alpha_{L})(k_{3}k_{6}k_{8}-k_{3}\omega_{L}\alpha_{H}\right. \\& \left. \quad -k_{8}\omega_{N}\alpha_{L})\right] /N^{*}(k_{3}k_{6}k_{8}-k_{3}\omega_{L}\alpha_{H}-k_{8}\omega_{N}\alpha_{L}) \\& \quad\times(k_{2}k_{5}k_{7}-k_{2}\omega_{L}\alpha_{H} -k_{7}\omega_{N}\alpha_{L})\\ \mathcal{R}_{H} &= \beta_{H}U^{*}_{H}\left[\alpha_{L}\alpha_{H}(k_{6}k_{8}+k_{8}\omega_{N}+\omega_{N}\omega_{L}-\omega_{L}\alpha_{H})\sigma_{N}\right.\\ &\quad +k_{2}\alpha_{H}(k_{3}k_{8}+k_{3}\omega_{L}+k_{8}\alpha_{L})\sigma_{L} \\ &\quad +(k_{3}k_{6}+k_{3}\alpha_{H}+\alpha_{L}\alpha_{H}-\omega_{N}\alpha_{L})(k_{2}k_{5}-\omega_{N}\alpha_{L})\sigma_{H} \\ &\quad \left.+(k_{2}k_{5}+k_{2}\alpha_{H}+\alpha_{L}\alpha_{H}-\omega_{N}\alpha_{L})(k_{3}k_{8}k_{6}-k_{8}\omega_{N}\alpha_{L}\right. \\& \quad \left.-k_{3}\omega_{L}\alpha_{H})\right] /N^{*}(k_{3}k_{6}k_{8}-k_{3}\omega_{L}\alpha_{H}-k_{8}\omega_{N}\alpha_{L})\\&\quad\times (k_{2}k_{5}k_{7}-k_{2}\omega_{L}\alpha_{H}-k_{7}\omega_{N}\alpha_{L}). \end{aligned} $$

Furthermore, the expression $\mathcal {R}_{N}$ is the number of secondary infections among the non-injection drug users, $\mathcal {R}_{L}$ is the number of secondary infections among the low-risk injection drug users, $\mathcal {R}_{H}$ is the number of secondary infections among the high-risk injection drug users. These expressions ($\mathcal {R}_{N}, \mathcal {R}_{L}, \mathcal {R}_{H}$) include the secondary infection in each sub-groups due to both horizontal and vertical transitions of infectious individuals due to disease translation and risky behaviors within and between the subgroups.

The basic reproduction number $\mathcal {R}_{0}$ is defined as the average number of new infections that is produced as a result of the introduction of one infectious individual into a population that is fully susceptible [16–19].

#### The basic reproduction number when *ω*_*N*_=*ω*_*L*_=*α*_*L*_=*α*_*H*_=0

Suppose the vertical upward and downward transition between the subgroups are absent, that is, *ω*_*N*_=0, *ω*_*L*_=0, *α*_*L*_=0, *α*_*H*_=0, then the basic reproduction number of the MRSA model (1) with IDUs, is given as:

3 $$\begin{array}{@{}rcl@{}} \mathcal{R}_{0} &=& \mathcal{R}_{N} + \mathcal{R}_{L} + \mathcal{R}_{H}, \end{array} $$

where

4 $$\begin{array}{@{}rcl@{}}  \mathcal{R}_{N}&=&\frac{\beta_{N} U^{*}_{N}(k_{3}+\sigma_{N})}{k_{2}k_{3}N^{*}},\quad \mathcal{R}_{L}~=~\frac{\beta_{L} U^{*}_{L}(k_{6}+\sigma_{L})}{k_{5}k_{6}N^{*}},\\ \mathcal{R}_{H}&=&\frac{\beta_{H} U^{*}_{H}(k_{8}+\sigma_{H})}{k_{7}k_{8}N^{*}}. \end{array} $$

It should be noted that the reproduction number stated in Eq. (2) gives the reproduction number in Eq. (3) in the absence of vertical downward and upward transition, that is, if *ω*_*N*_=*ω*_*L*_=*α*_*L*_=*α*_*H*_=0.

### Parameter estimation

#### MRSA Demographic data from 2008-2013

**Fig. 2:**
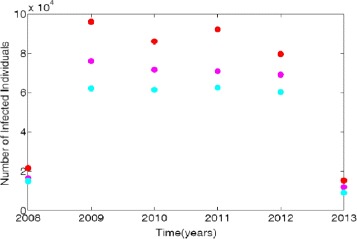
Demographic data from three regional areas, large metro, large suburb, and rural. Red dots represent large metro areas, magenta correspond to the large suburb areas and turquoises blue represent the data for rural areas

Demographic data from 2008-2013 was obtained from the Agency for Healthcare Research and Quality (AHRQ) [20]. The AHRQ compiles international classification of diseases (ICD) data from hospitals throughout the United States. These data include patients who were discharged with MRSA and are identified with ICD-9 code. To obtain the MRSA ICD-9 data, AHRQ demographic data targets were set to look at large metro, large suburb, and rural areas. As expected, the large metro population produced the highest number of patients with MRSA listed on their medical records upon discharge. Figure 2 displays the obtained data in tens of thousands of hospital discharges from 2008-2013.

#### Non-IDU subgroup parameter estimation

In order to parametrize MRSA model (1) we use patient data obtained from the AHRQ surveys. One of the limitations of this data is that it does not identify individuals with MRSA that are injection drug users. To overcome this short coming, we first assume that the population consist of only non-IDUs and then we reduce the MRSA model (1) to the following system of ordinary differential equations

5 $$\begin{array}{@{}rcl@{}} \frac{dU_{N}}{dt} &=& \pi_{N} + \gamma_{N} I_{N} + \tau_{N} C_{N} - \frac{\beta_{N} (C_{N}+I_{N})}{N} - \mu U_{N} \\ \frac{dC_{N}}{dt} &=& \frac{\beta_{N} (C_{N}+I_{N})}{N} - (\tau_{N}+\sigma_{N}+\mu) C_{N} \\ \frac{dI_{N}}{dt} &=& \sigma_{N} C_{N} - \left(\gamma_{N}+\mu+\delta_{N}\right)I_{N} \end{array} $$

For the reduced MRSA model (5) with non-IDUs, the total population *N*=*U*_*N*_+*C*_*N*_+*I*_*N*_, and the force of infection is given by $\frac {\beta _{N} (C_{N}+I_{N})}{N}$. The basic reproduction number is given as:

6 $$\begin{array}{@{}rcl@{}}  \mathcal{R}_{N}&=&\frac{\beta_{N} U^{*}_{N}(k_{3}+\sigma_{N})}{k_{2}k_{3}N^{*}}, \end{array} $$

where *k*_1_=*μ*, *k*_2_=*τ*_*N*_+*σ*_*N*_+*μ*, *k*_3_=*γ*_*N*_+*μ*+*δ*_*N*_.

**Fig. 3:**
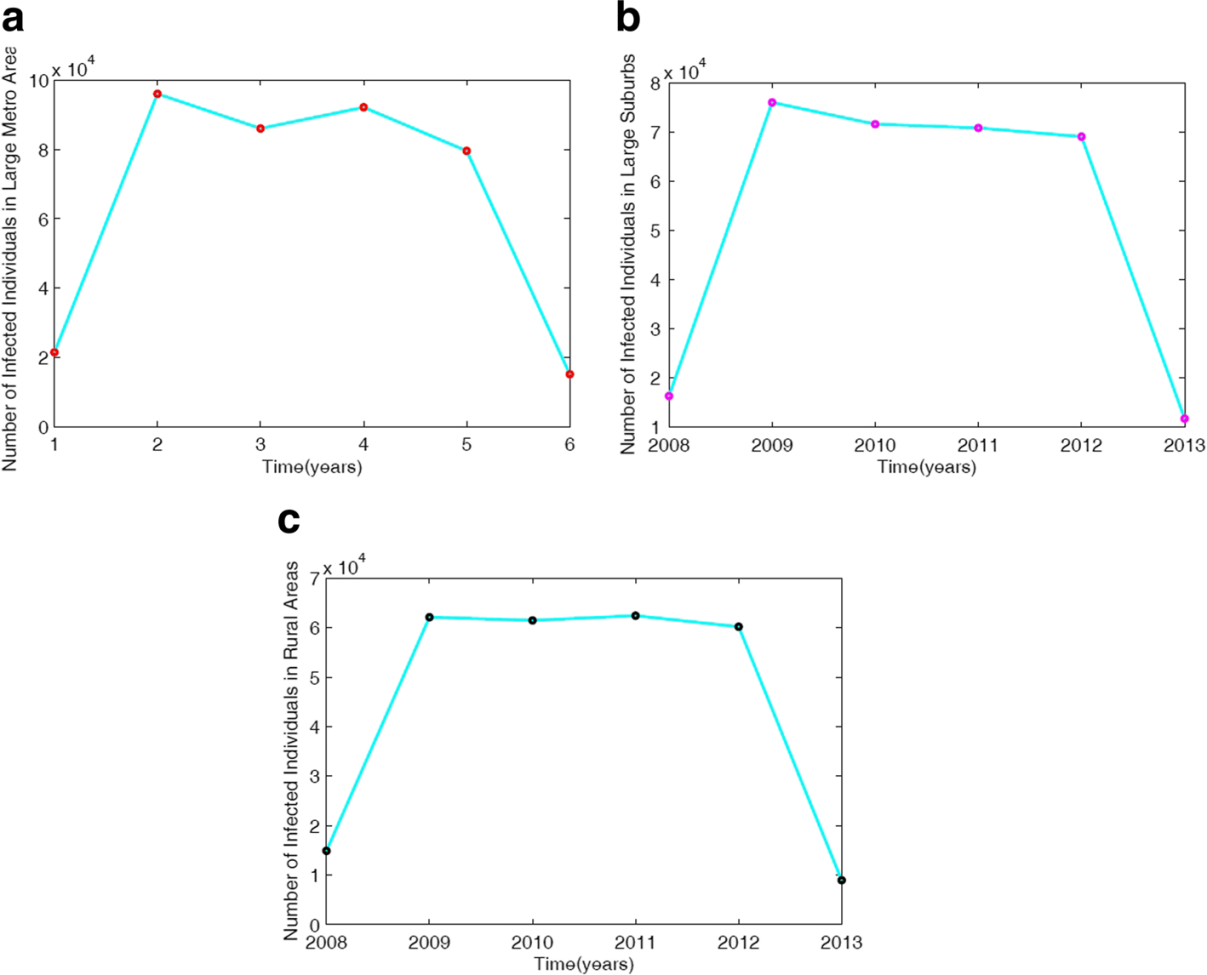
Simulation of the non-IDUs reduced MRSA model (5) fitted to the ICD-9 MRSA data for: (**a**) Large metro area data and model simulation; (**b**) Large suburbs area data and model simulation; (**c**) Rural area data and model simulation. Turquoise solid lines represent model simulations, red dot in (**a**) represent large metro area data, magenta dot in (**b**) is the data for large suburb area and the black dot in (**c**) is the data for rural area

Next, we estimate the parameters of the reduced MRSA model (5) by using the ICD-9 MRSA data for large metro, rural, and suburb regional areas. The results of the parameter estimation are given in Table 2; the model simulation profile and the fitted data are depicted in Fig. 3.

**Table 2:** Parameter estimation of the non-injection drug users using the reduced MRSA model (5) and the regional data obtained from the Agency for Healthcare Research and Quality (AHRQ) [20]

Variable	Large metro	Rural	Suburb
*β*_*N*_	0.2895	0.2015	0.1444
*γ*_*N*_	0.2778	0.1874	0.1316
*σ*_*N*_	0.7079	0.6670	0.6560

A number of studies have identified injection drug use as a significant risk factor for developing MRSA secondary to hospitalization rates and increased exposure to antibiotics arising from treatment of skin abscesses, bacteremias, and endocarditis [12–14]. Cohen [21] established a relationship between the use of methamphetamine and the occurrence of MRSA. Hence, we assume that the relationship between injection drug use and the risk of developing MRSA is linear. This enabled us project the non-IDUs parameters onto the low- and high-risk IDUs parameters using some risk related parameters discussed below.

Therefore, in order to obtain the parameter estimates for the IDUs in the full MRSA model (1), we modify the transmission probability, disease progression and recovery rates in the low- and high-risk subgroups by multiplying the non-IDUs subgroup parameter estimates *β*_*N*_,*σ*_*N*_, and *γ*_*N*_ with *ε*_*L*_ and *ε*_*H*_. These parameters represent the associated risky behaviors in low-risk and high-risk subgroups. The non-IDU disease induced mortality *δ*_*N*_ is also modified by *ε*_*L*_ and *ε*_*H*_, since IDUs have a higher mortality rate due to their drug use [22–24].

These modification parameters *ε*_*L*_ and *ε*_*H*_ are called risk factors; their values give us an estimate away (either in an increasing or decreasing form) from the non-IDUs parameters. How these parameter values are obtained for the IDUs in the low-risk, and high-risk subgroups are discussed below.

#### Risk factors *ε*_*L*_ and *ε*_*H*_ formulation

The risk factor is designed to distinguish the parameter values in all the subgroups from one another. It incorporates qualitative characteristics of injection drug use in the low-risk and high-risk subgroup transformed into quantitative integers that are used to impact the low-risk and high-risk parameters described in the MRSA model (1) formulation.

Hence, the low-risk IDU subgroup transmission probability (*β*_*L*_), disease progression rate (*σ*_*L*_), recovery rate (*γ*_*L*_), and MRSA-induced death (*δ*_*L*_) are all modified from the non-injection drug users parameter values by a risk factor (*ε*_*L*_) associated with the risk-associated behaviors. Hence,

7 $$\begin{array}{@{}rcl@{}} \beta_{L} &=& \beta_{N}(1+\varepsilon_{L}), \quad\sigma_{L} = \sigma_{N}(1+\varepsilon_{L}), \\ \gamma_{L} &=& \gamma_{N}(1-\varepsilon_{L}),\quad \delta_{L} = \delta_{N}(1+\varepsilon_{L}). \end{array} $$

The parameters *β*_*L*_ and *σ*_*L*_ are both increased by the introduction of a risk factor *ε*_*L*_. This increases the rate at which individuals move from the uncolonized class, *U*_*L*_, to the colonized class, *C*_*L*_, and subsequently, to the infected class, *I*_*L*_, compare to the individuals in the non-IDUs subgroup. On the other hand, *γ*_*L*_ is decreased by the effect of *ε*_*L*_, thus decreasing recovery rate and holding individuals in the infected compartment for a longer period compare to non-IDUs.

The high-risk IDU subgroup parameters undergo an analogous treatment but with a larger, and higher impacting value *ε*_*H*_ in place of *ε*_*L*_ as shown in [6]. Thus,

8 $$\begin{array}{@{}rcl@{}} \beta_{H} &=& \beta_{N}(1+\varepsilon_{H}), \quad\sigma_{H} = \sigma_{N}(1+\varepsilon_{H}), \\ \gamma_{H} &=& \gamma_{N}(1-\varepsilon_{H}), \quad\delta_{H} = \delta_{N}(1+\varepsilon_{H}). \end{array} $$

To estimate the values of the risk factors *ε*_*L*_ and *ε*_*H*_, we first construct an ethogram. The practice of using ethograms is common within the study of animal behavior [25, 26]. This technique allows the researcher to quantify behaviors observed in the subject(s) and their interaction with other organism or with their environment. This technique was used to construct Tables 3 and 4.

**Table 3:** Ethogram displaying and quantifying injection drug users risk associated behaviors

Ranking	Classification	Definition
0	No drug use	No use of injection drugs
1	Limited drug use	Lone occasional use of injection drugs
2	Mild drug use	Occasional use of injection drugs with other users and visiting shooting galleries
3	Moderate drug use	Sharing/ splitting drugs
4	Intense drug use	Sharing/ splitting drug paraphernalia
5	Severe drug use	Occupying a shooting gallery full time

**Table 4:** Ethogram complementary table displaying and quantifying injection drug user risk associated behaviors

Rating	Class assignment	Risk factor (%)
0	Non-injection drug user	0
1-6	Low-risk injection drug user	6-36
7-15	High-risk injection drug user	42-90

To construct Table 3, we classify individuals’ associated risk behavior from non-drug use to severe drug use, assigning a value from 0-5 based on the perceived level of risk. The ranking values in Table 3 is then translated to the rating points in the complimentary Table 4 by summing the ranking values of an injection drug user from their previous to current categories. For instance, a moderate IDU has a rating point of 6; this comes from them having been a limited to mild and moderate drug user (i.e., 0+1+2+3=6, obtained from ranking values in Table 3). Thus, a severe IDU will have a ranking of 15 points (i.e., 0+1+2+3+4+5+6=15).

Using these ranking points, we classify the individuals into low-risk and high-risk IDUs (see Table 4). To obtain the risk factor percentages, we use a ratio of 1 rating point to 6% risk factor value (1 rating = 6% risk factor); the ratio of 1-6 is chosen since there are six risk categories under consideration. Thus, we have 6-36% for a low-risk IDU and a 42-90% high-risk IDUs thresholds. These represent the percentage that the non-IDU parameters will be amplified by (*β*_*N*_,*σ*_*N*_,*δ*_*N*_) or decreased by (*γ*_*N*_) due to associated IDU risky behaviors.

### Sensitivity analysis

In order to determine the robustness of the model in relation to each parameter, a sensitivity analysis was performed. Analysis of parameters can give insight into the uncertainty an input may have and how this will affect the outcome of the model. To determine system sensitivity to its parameters, the normalized forward sensitivity index [27–29] given in Eq. (9) is used

9 $$\begin{array}{@{}rcl@{}} Y^{\mathcal{R}_{N}}_{p} &=& \frac{\partial \mathcal{R}_{N}}{\partial p} \times \frac{p}{\mathcal{R}_{N}}, \end{array} $$

where *p* represent the parameter of interest and $\mathcal {R}_{N}$, the reproduction number $\mathcal {R}_{N}$ of the reduce model (5); since the parameters of MRSA model (1) were obtained from fitting to data the reduced model (5) and modifying their values using the risk factors *ε*_*L*_ and *ε*_*H*_ to obtain the values for the IDUs parameters. Hence, the local sensitivity analysis was performed on the following parameters: the transmission probability (*β*_*L*_), disease progression rate (*σ*_*L*_), recovery rate (*γ*_*L*_), MRSA-induced death (*δ*_*L*_), and natural death (*μ*) based on their influence on $\mathcal {R}_{N}$.

## Control measures

The best strategy to preventing MRSA infections among IDUs would be for them to end their risky behaviors, many of these IDUs are not ready to stop or make a change in their behaviors [30]. However, by simply learning new hygiene skills or best practices may substantially decrease the risk of MRSA among individuals who are reluctant to stop injecting drugs [30, 31]. For instance, Hart et al. observed a reduction in the incidence of abscesses among IDUs registered in a needle exchange program in London.

In this section we considered two types of control strategies; the first strategy investigates the impact of vertical downward transition between the subgroups due to individuals in the community engaging in risky behaviors that promote injection drug use and the impact of vertical upward transition that can be achieved by enrolling in rehabilitation programs. The aim of this strategy is to investigate the impact of the parameters *ω*_*N*_ and *ω*_*L*_, for the vertical downward transition and the parameters *α*_*L*_ and *α*_*H*_ for the vertical upward transition.

The second strategy uses results obtain from the sensitivity analysis to consider control within each subgroup. With this strategy, we target the parameters *β*_*N*_ and *γ*_*N*_ by implementing three different control strategies: low-effectiveness strategy, moderate-effectiveness strategy, and high-effectiveness strategy. The goal of each strategy is to reduce *β*_*N*_ and increase *γ*_*N*_. The risk factors *ε*_*L*_ and *ε*_*H*_ are then used to determine the values of *β*_*L*_, *β*_*H*_, *γ*_*L*_ and *γ*_*H*_ in the low-and high-risk IDUs subgroups respectively.

Note that for the two different control strategies either between or within the subgroups, we used the intermediate risk factor values *ε*_*L*_=21% and *ε*_*H*_=66 %.

### Control of risky injection drug use behaviors

In this section, we investigate the impact of vertical downward and upward transitions between the compartments due to risky behaviors that promotes (downward transitions) and discourages (upward transitions) injection drug use in the community. The aim of this control strategy is to investigate the impact of vertical downward transition using the parameters *ω*_*N*_ and *ω*_*L*_, and impact of the vertical upward transition using the parameters *α*_*L*_ and *α*_*H*_. Note in this section we are controlling the risky behaviors and not MRSA transmission within the subgroups.

#### No vertical downward and upward transitions

First, suppose that there are no transition between the subgroups (i.e., non-IDUs, low-risk IDUs and high-risk IDUs); in other words, there are no vertical downward transitions due to risky behaviors nor are there vertical upward transitions due to enrollment in rehabilitation programs, that is *ω*_*N*_=0,*ω*_*L*_=0,*α*_*L*_=0,*α*_*H*_=0.

#### Vertical downward transitions only

Next, we consider the situation with only vertical downward transitions and no upward transitions. Suppose individuals in the non-IDUs slowly engage in the risky behavior of injection drug use, but individuals in the low-risk IDUs quickly engage in these risky behaviors either due to peer pressure or due to individuals physiology [32, 33]. Thus, we set *ω*_*N*_=0.05825,*ω*_*L*_=0.116,*α*_*L*_=0,*α*_*H*_=0.

#### Vertical upward transitions only

Next, we investigate the impact of the vertical upward transitions that can be achieved by enrolling in rehabilitation programs [3, 6]. Suppose more individuals in the high-risk IDUs enter rehabilitation programs either due to referrals from those around them or due to increased access to such programs [34] or through family interventions [35]. Further, suppose fewer low-risk IDUs enter rehabilitation program because they either do not see the need for the program or they believe they could handle the problem without the rehabilitation program or they believe in their ability to control their risky behaviors [36]. For this scenario, we set *ω*_*N*_=0,*ω*_*L*_=0,*α*_*L*_=0.0112,*α*_*H*_=0.0560.

#### Vertical downward and upward transitions

Lastly, we investigate the impact of both downward and upward transitions due to changes in risky behaviors; that is, we set *ω*_*N*_=0.05825,*ω*_*L*_=0.116,*α*_*L*_=0.0112,*α*_*H*_=0.0560.

### Control of MRSA transmission among the sub-groups

According to the Centers for Disease Control and Prevention (CDC), maintaining adequate personal hygiene is vital for the control of MRSA in the community [37]. In hospital setting, health-care workers are required to constantly clean their clothing, laundry, medical equipment and the entire hospital environment to prevent and minimize contact and transmission of the bacteria [37].

In this section, we investigate the impact of controlling MRSA transmission within the subgroups, we will not consider decolonization as a control strategy. Thus, to determine the impact of controlling MRSA within the subgroups, we use results obtained from the sensitivity analysis. From the sensitivity analysis, we observed that control strategies that reduce *β*_*N*_ and increases *γ*_*N*_ would impact MRSA in the community since these parameters have strong positive (*β*_*N*_) and negative (*γ*_*N*_) impact on the reproduction number $\mathcal {R}_{N}$. The same holds for the parameters *β*_*L*_,*β*_*H*_,*γ*_*L*_, and *γ*_*H*_. Hence, we implement three different strategies: low-effectiveness strategy, moderate-effectiveness strategy, and high-effectiveness strategy. Note that these strategies are only for theoretical purpose to illustrate the impact of these control interventions.

#### Low-effectiveness control strategy

Our aim in this section is to investigate the control strategies that reduces MRSA transmission in the community. To achieve this in both the non-IDUs and IDUs populations, we assumed that the IDUs do not forfeit any of their risk associated behaviors. And they also do not alter their medical circumstances by actively seeking out medical treatment.

Thus, for this low-effective strategy, we set *β*_*N*_=0.27780 and *γ*_*N*_=0.27780 (the values obtained from the data fitting above). Using the modifying risk factors *ε*_*L*_ and *ε*_*H*_, we obtain the values for *β*_*L*_,*γ*_*L*_,*β*_*H*_ and *γ*_*H*_ respectively. That is, *β*_*L*_=*β*_*N*_(1+*ε*_*L*_),*β*_*H*_=*β*_*N*_(1+*ε*_*H*_),*γ*_*L*_=*γ*_*N*_(1−*ε*_*L*_), and *γ*_*H*_=*γ*_*N*_(1−*ε*_*H*_).

#### Moderate-effectiveness control strategy

We reiterate that unlike in the previous section, our aim in this section is not to control the risky behavior but to control the transmission of MRSA within the subgroups. Thus, to implement the moderate-effectiveness control strategy, we assume that if an IDU ceases to share their drug paraphernalia with other IDUs [6], this will reduce the physical contact with other IDUs which invariably reduces the transmission probabilities *β*_*N*_, *β*_*L*_, and *β*_*H*_.

Hence, for the moderate-effectiveness control strategy we set

$$\begin{array}{ll} \beta_{N} = \beta_{N}/2,& \gamma_{N} \ =\ (1+0.007)\gamma_{N}\\ \beta_{L} = \beta_{N}(1+\varepsilon_{L}), &\beta_{H}\ =\ \beta_{N}(1+\varepsilon_{H})\\ \gamma_{L} = \gamma_{N}(1-\varepsilon_{L}), & \gamma_{H}\ =\ \gamma_{N}(1-\varepsilon_{H}). \end{array} $$

Note that recovery rate (*γ*_*N*_) in the non-IDUs have been increased by 0.7*%*.

#### High-effectiveness control strategy

We assume that the IDUs in the community enroll in a drug abuse program (DAP) [3, 6]. Note that the voluntary or involuntary enrollment into a DAP will not stop the use of injection drugs, but it will significantly decrease the practice of risk-associated behaviors. Education and accountability given to individuals enrolled in the DAP will effectively reduce the risk-associated behaviors listed in Table 3 and by extension will lead to a reduction in the disease transmission probabilities *β*_*N*_, *β*_*L*_, and *β*_*H*_ [5, 6].

Additional benefits gained by the IDUs who participate in the program include access to medical professionals and to resources such as symptom and treatment education. These benefits can empower MRSA infected IDUs with tools necessary to recognize and treat their infections. Bassetti et al. found that the addition of medical care to MRSA infected IDU patients significantly reduced the use of drugs [3]. As explained in [3], a major benefit of DAPs is access to quality health care. Such access will not only provide a better understanding of disease transmission and hygiene techniques but will also provide timely diagnosis and a more precise consumption of medicine. This improved access to health care invariably leads to increase in the recovery rate of the IDUs, that is, increase in *γ*_*L*_ and *γ*_*H*_.

Thus, for the high-effectiveness control strategy we set

$$\begin{array}{ll} \beta_{N} = \beta_{N}/4, & \gamma_{N} = (1+0.009)\gamma_{N}\\ \beta_{L} = \beta_{N}(1+\varepsilon_{L}), & \beta_{H} = \beta_{N}(1+\varepsilon_{H})\\ \gamma_{L} = \gamma_{N}(1-\varepsilon_{L}), & \gamma_{H} = \gamma_{N}(1-\varepsilon_{H}). \end{array} $$

In this case, the recovery rate (*γ*_*N*_) in the non-IDUs have been increased by 0.9*%*.

## Results

In this paper, we have designed and studied a deterministic model for methicillin-resistant Staphylococcus aureus (MRSA) transmission in the community with injection drug users (IDUs). Unique to our model is the incorporation of two IDUs with low- and high-risk associated behaviors. Our goal in this paper is to understand the transmission dynamics of MRSA in a community with IDUs and to investigate the impact of different control strategies in curtailing MRSA transmission in the community. Thus, the intervention strategies analyzed include the impact of drug rehabilitation, intervention and education, and clean needle exchange programs on MRSA transmission in the community (i.e., the vertical upward and downward transition) as well as three control effectiveness strategies at each subgroup levels (i.e., low-effectiveness, moderate-effectiveness, and high-effectiveness strategies).

The model theoretical results indicate that the disease-free equilibrium of the model is locally-asymptotically stable whenever the related reproduction number is less than unity and stable otherwise. The implication of this result is that the disease will die out or be curtailed whenever the reproduction number is less than unity and will spread whenever the reproduction is greater than unity.

### Parameter estimation

We parameterized the model using the 2008-2013 Agency for Healthcare Research and Quality (AHRQ) data-set; these data include patients who were discharged with MRSA. A major limitation with the use of this data-set is that it includes individuals who may have acquired MRSA infection due to their hospital stay. As a result, the data-set does not truly capture the transmission of the bacteria in the community which is more appropriate for evaluating MRSA burden amongst IDUs in the community. To overcome this limitation, we assumed that the population consist of only non-IDUs and reduce the MRSA model (1). This enabled us determine parameters that are projected onto the low and high risk-IDUs using risk factors *ε*_*L*_ and *ε*_*H*_.

**Table 5:** Sensitivity Index using parameter estimates obtained from the reduced MRSA model (5)

Variable	Sensitivity index
*β*_*N*_	1.0000
*γ*_*N*_	-0.7164
*σ*_*N*_	-0.2819
*μ*	-0.0008
*δ*_*N*_	-0.0009

The result shows that *β*_*N*_ and *γ*_*N*_ have the greatest impact on the output value of $\mathcal {R}_{N}$

Having obtained the estimate for the risk factors *ε*_*L*_ and *ε*_*H*_, we investigated the impact of varying these modification parameters on disease transmission in the community. For the low-risk factor *ε*_*L*_, we used the lower and upper bound values (6% and 36%) given in Table 4 and their intermediate value (21%). Similarly, for the high-risk factor, we used 42%, 66%, and 90%.

**Fig. 4:**
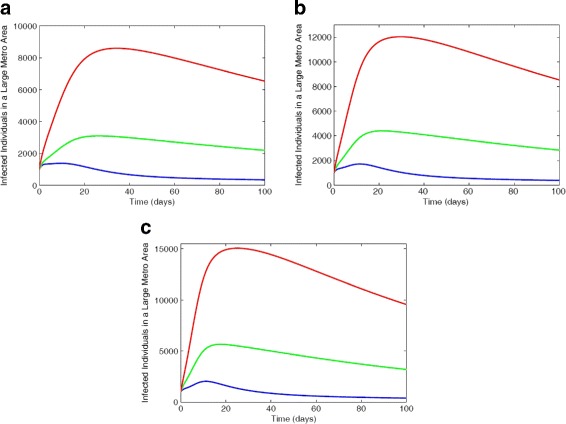
Simulation of the MRSA model (1) with varied risk factors *ε*_*L*_ and *ε*_*H*_ values. **a** Infected individuals with 6% lrf and 42% hrf. **b** Infected individuals with 21% lrf and 66% hrf. **c** Infected individuals with 36% lrf and 90% hrf. Parameter values used are as given in Tables 6 and 7. Blue lines correspond to non-IDUs, green represent the low-risk IDUs and red represent the high-risk IDUs

Figure 4 depict the results of simulating the full MRSA model (1) using the parameter estimates given in Table 2 and the low, intermediate, and high bounds of the risk factors *ε*_*L*_ and *ε*_*H*_ in Table 4. At the lower bounds of the risk factors (*ε*_*L*_=6% and *ε*_*H*_=42%), the non-IDUs have the highest number of colonized individuals, this is followed by low-risk IDUs, while the high-risk IDUs have the least number of colonized individuals (not shown here in the figure). Within the infected compartment, the high-risk IDUs have the highest number of individuals followed by the low-risk then non-IDUs. The disparity between each subclass increases when the risk factor is increased such as the high-risk IDUs with a greater number of infected compared to the low-risk and non-IDUs.

**Table 6:** Parameter values and ranges for MRSA model (1) with IDUs

Variable	Baseline value	References
*π*_*N*_	0.01250	[49]
*π*_*L*_	0.00625	Estimated
*π*_*H*_	0.00313	Estimated
*β*_*N*_	0.28950	Fitted
*σ*_*N*_	0.70790	Fitted
*γ*_*N*_	0.27780	Fitted
*τ*	0.09000	[9]
*ω*_*N*_	0.05825	Fitted
*ω*_*L*_	0.11650	[50]
*α*_*L*_	0.11200	Estimated
*α*_*H*_	0.05560	[51]
*μ*	0.00824	[52, 53]

**Table 7:** Parameter values and ranges for MRSA model (1) with IDUs using the risk factors *ε*_*L*_ and *ε*_*H*_ ranges given in Table 4

Variable	Baseline value	Range	References
*β*_*L*_	0.30500	(0.30687-0.39372)	Estimated
*β*_*H*_	0.48060	(0.41109-0.55002)	Estimated
*σ*_*L*_	0.85656	(0.75047-0.96274)	Estimated
*σ*_*H*_	1.17511	(1.00522-1.34501)	Estimated
*γ*_*L*_	0.21946	(0.26113-0.17779)	Estimated
*γ*_*H*_	0.09445	(0.16110-0.02778)	Estimated
*ε*_*L*_	0.21000	(0.06000-0.36000)	Estimated
*ε*_*H*_	0.66000	(0.42000-0.90000)	Estimated
*δ*_*N*_	0.00033	(0.48060-0.55010)	[53]
*δ*_*L*_	0.00040	(0.00035-0.000045)	Estimated
*δ*_*H*_	0.00047	(0.00055-0.00063)	Estimated

**Fig. 5:**
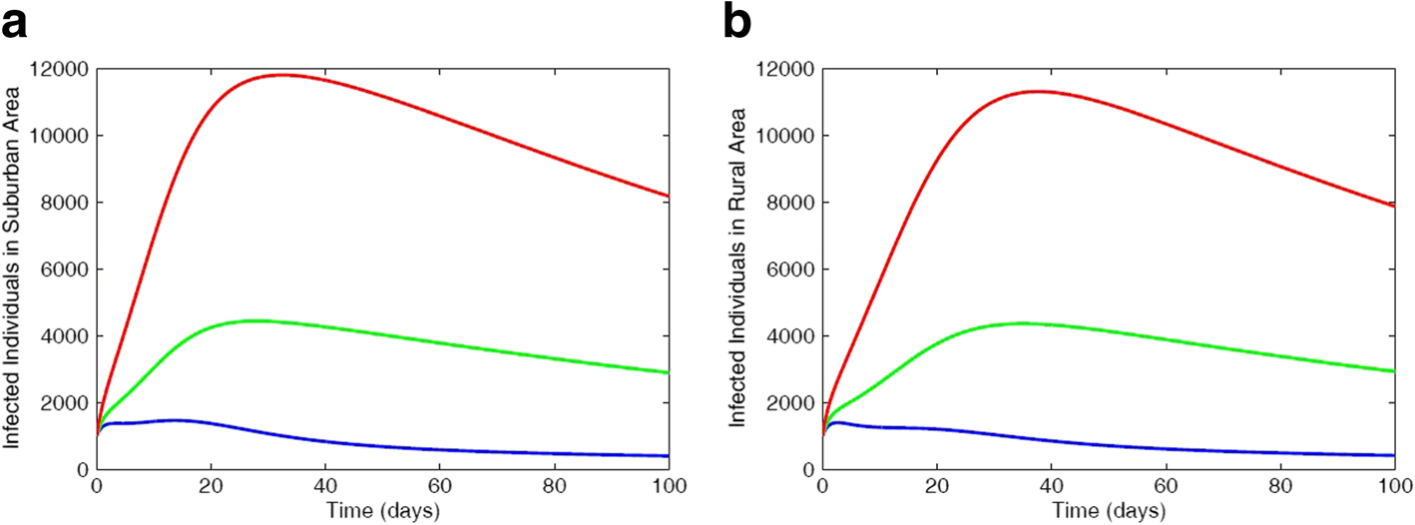
Simulation of the MRSA model using regional data with 21% low-risk factor and 66% high-risk factor. **a** Infected individuals in suburb area. **b** Infected individuals in rural area. Parameter values used are as given in Tables 6 and 7. Blue lines correspond to non-IDUs, green represent the low-risk IDUs and red represent the high-risk IDUs

A similar trend is observed in Fig. 5 for the suburb and rural regional areas using intermediate risk factors estimates *ε*_*L*_=21% and *ε*_*H*_=66%.

For the rest of the paper, we will use the parameter estimates for the large metro area since the number of patients in the ICD-9 data for this area is the largest; moreover, the results for the large suburb and rural areas are not expected to deviate from the results obtained for large metro areas.

### Sensitivity analysis

The outcome of the local sensitivity analysis using parameters estimated in Table 2 is shown in Table 5; the parameter *β*_*N*_ and *γ*_*N*_ are both shown to have the largest impact on the reproduction number ($\mathcal {R}_{N}$). The implication of this result is that any control strategy which target these two parameters will give the greatest impact on $\mathcal {R}_{N}$. For instance a control strategy that decreases *β*_*N*_ by 10% will lead to a 10% reduction in $\mathcal {R}_{N}$, similarly, a strategy that increases *γ*_*N*_ by 10% will lead to a 7.2% decrease in $\mathcal {R}_{N}$. In the next section, we addressed control measures that targets *β*_*N*_ and *γ*_*N*_ with the goal of reducing $\mathcal {R}_{N}$.

### Control measures

We investigated the impact of the different control strategies to curtail MRSA transmission within and between each subgroup in the community by first controlling the risky behaviors within the groups and secondly by controlling the transmission of the bacteria among the groups using parameter values given in Tables 6 and 7.

### Control of risky injection drug use behaviors

Under this control strategy, we consider four scenarios involving transitions between the groups.

#### No vertical downward and upward transitions

**Fig. 6:**
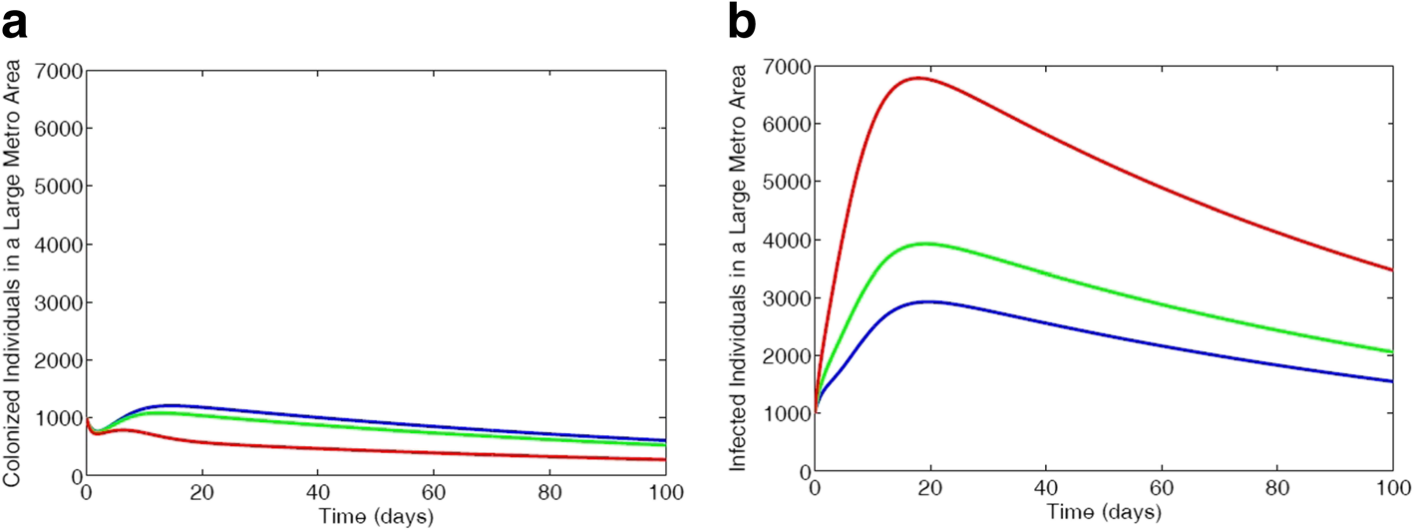
MRSA model simulation with large metro demographics. Each simulation shows a restriction of individuals transition between sub-groups with the parameter values *ω*_*N*_=0,*ω*_*L*_=0,*α*_*L*_=0,*α*_*H*_=0 (**a**) Colonized individuals, (**b**) Infected individuals. Parameter values used are as given in Tables 6 and 7. Blue lines correspond to non-IDUs, green represent the low-risk IDUs and red represent the high-risk IDUs

We observed in Fig. 6a that the non-IDUs have more colonized individuals followed by the low-risk IDUs and the high-risk IDUs have the least number of colonized individuals. These dynamics is due to the fact that disease progression rate is highest in the high-risk IDUs and smallest in the non-IDUs. This explains the result of Fig. 6b where the high-IDUs have the most number of infected individuals followed by the low-risk IDUs, and the non-IDUs have the least number of infected individuals.

#### Vertical downward transitions only

**Fig. 7:**
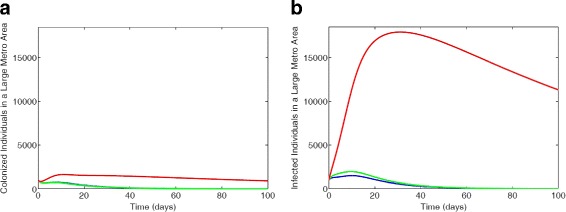
MRSA model simulation with large metro demographics. Each simulation shows a restriction of individuals transition between sub-groups with the parameter values *ω*_*N*_=0.05825,*ω*_*L*_=0.116,*α*_*L*_=0,*α*_*H*_=0 (**a**) Colonized individuals, (**b**) Infected individuals. Parameter values used are given in Tables 6 and 7. Blue lines correspond to non-IDUs, green represent the low-risk IDUs and red represent the high-risk IDUs

We observed in Fig. 7 an increase in the number of colonized and infected high-risk individuals and a decrease in the colonized and infected compartments of the other two subgroups. The result of this simulation can be thought of as a funnel that sifts individuals down through the model into more risky behaviors and thus an increase in transmission of MRSA for the high-risk IDUs.

#### Vertical upward transitions only

**Fig. 8:**
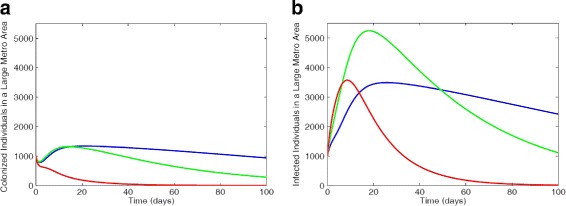
MRSA model simulation with large metro demographics. Each simulation shows a restriction of individuals transition between sub-groups with the parameter values *ω*_*N*_=0,*ω*_*L*_=0,*α*_*L*_=0.0112,*α*_*H*_=0.0560 (**a**) Colonized individuals, (**b**) Infected individuals. Parameter values used are as given in Tables 6 and 7. Blue lines correspond to non-IDUs, green represent the low-risk IDUs and red represent the high-risk IDUs

We observed more colonized individuals in the non-IDUs subgroup in Fig. 8a, followed by the low-risk IDUs and the high-risk IDUs have the least number of colonized individuals. However, in the infected compartment (see Fig. 8b) the low-risk IDUs have more individuals for about 55 days due to the surge from the high-risk individuals changing their behavior. But over time, the non-IDUs have more infected individuals as individuals move upwards between the subgroups.

#### Vertical downward and upward transitions

**Fig. 9:**
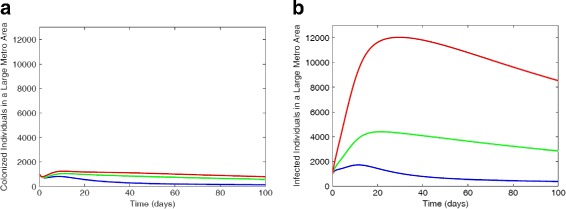
MRSA model simulation with large metro demographics. Each simulation shows a restriction of individuals transition between sub-groups with the parameter values *ω*_*N*_=0.05825,*ω*_*L*_=0.116,*α*_*L*_=0.0112,*α*_*H*_=0.0560 (**a**) Colonized individuals, (**b**) Infected individuals. Parameter values used are as given in Tables 6 and 7. Blue lines correspond to non-IDUs, green represent the low-risk IDUs and red represent the high-risk IDUs

We observed in Fig. 9 similar effect as seen in Fig. 7 but in this case, fewer non-IDUs individuals were colonized and infected; this is due to the transitions to the other two subgroups. Thus, there are more individuals in the colonized and infected compartments in the high-risk, and low-risk IDUs subgroups in Fig. 9 compare to those in Fig. 7.

Hence, we have explored in this section the impact of the vertical downward and upward transitions among non-injection drug users and injection drug users who are both low-and high-risk users due to change in risky behaviors and found that as individual engage in these risky behaviors, MRSA cases in the community increase. However, with more IDUs enrolling into rehabilitation, intervention, and education, and clean needle exchange programs MRSA cases reduces in the community. This control is intended to simulate the decline in risky behavior which could be achieved by rehabilitation programs, intervention and education, clean needle exchange programs, and so forth [7, 8].

### Control of MRSA transmission among the sub-groups

The result of the sensitivity analysis was used to study the horizontal translation within the subgroups due to disease transmission by implementing three different strategies: low-effectiveness strategy, moderate-effectiveness strategy, and high-effectiveness strategy with the goal of reducing the number of colonized and infected individuals.

**Fig. 10:**
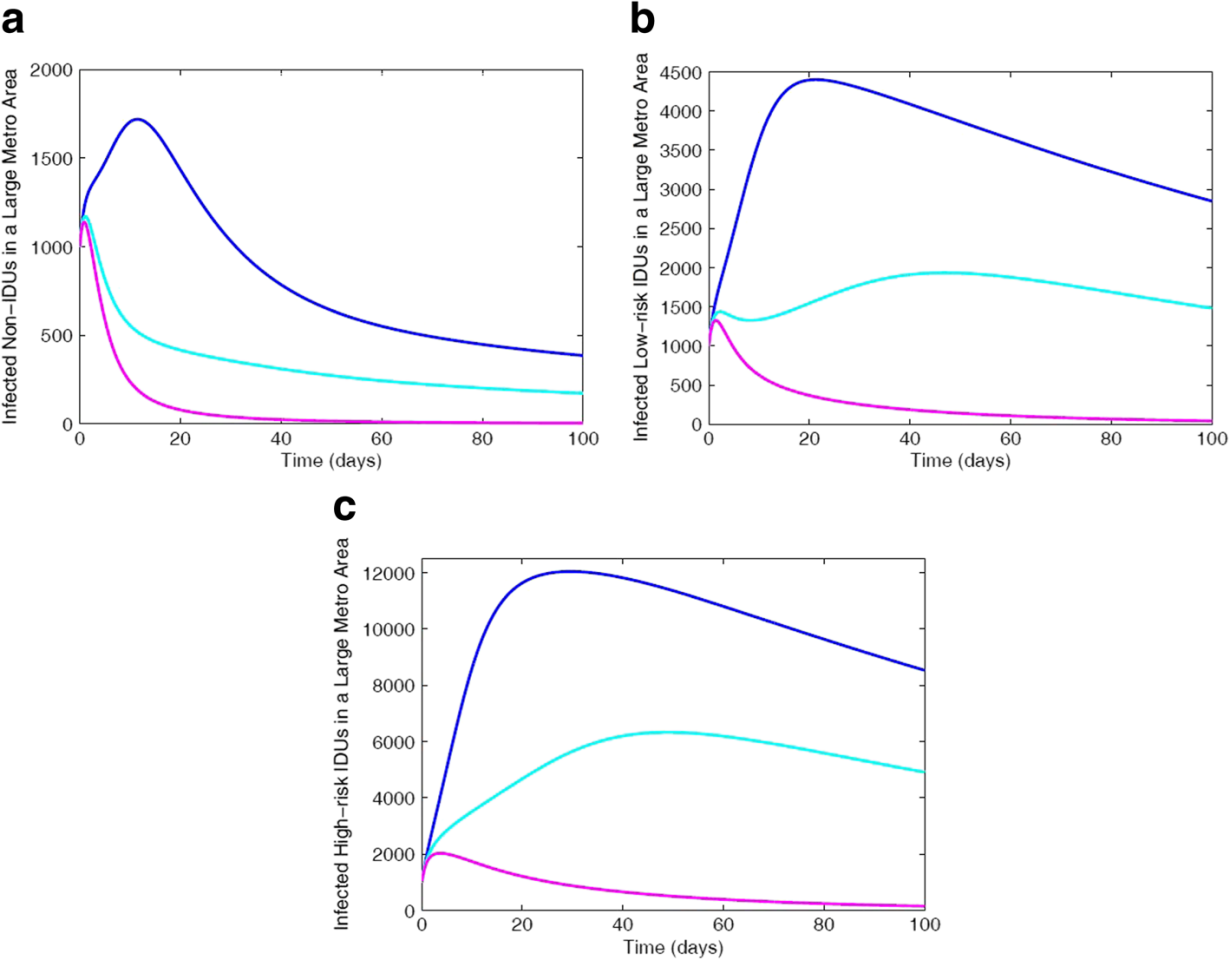
Simulation of the MRSA model (1) with IDUs showing the various effectiveness control strategies for total number of infected in each of the different subgroups: **a** Non-IDUs; **b** Low-risk IDUs; **c** High-risk IDUs. Parameter values used are as given in Tables 6 and 7. Blue lines correspond to low-effectiveness strategy, turquoise blue represent moderate-effectiveness strategy and magenta represent the high-effectiveness strategy

The total number of colonized and infected individuals in each of the different subgroups is simulated for the three levels of effectiveness for the low-effectiveness, moderate-effectiveness, and high-effectiveness control strategy and depicted in Fig. 10 are the solution profiles for the infected.

Figure 10 shows a reduction in each of the subgroups under the moderate- and high-effectiveness levels of the control strategy. It is worth noting that the high-effectiveness level of the control strategy, as expected, is far more effective in curtailing MRSA burden in the community. The moderate-effectiveness level of the strategy also resulted in a significant decline in the number of cases in comparison to the low-effectiveness level of the control strategy.

Specifically, the comparison of the three effectiveness strategies at *t*=100 days in each of the different subgroups (see Table 8), shows that the high-effectiveness control strategy led to a considerable reduction in the total number of colonized and infected individuals. This is followed by the moderate-effectiveness level, and the low-effectiveness level which produced the most number of colonized and infected cases.

**Table 8:** Simulation of the MRSA model (1) with IDUs showing the various effectiveness control strategies for total number of infected in each of the different subgroups at time *t*=100

Cases	Low-effective control	Moderate-effective control	High-effective control
Non-IDUs colonized cases	3.404526×10^5^	1.285701×10^5^	2.854360×10^4^
Low-risk IDUs colonized cases	7.949563×10^5^	3.411524×10^5^	4.270152×10^4^
High-risk IDUs colonized cases	1.025625×10^6^	5.593364×10^5^	6.303055×10^4^
Non-IDUs infected cases	8.330656×10^5^	3.404935×10^5^	9.403137×10^4^
Low-risk IDUs infected cases	3.582918×10^6^	1.687638×10^6^	2.544196×10^5^
High-risk IDUs infected cases	1.007395×10^7^	5.287627×10^6^	7.160370×10^5^

Thus, in this section, we found that the high-effectiveness control strategy is more effective in curtailing MRSA burden in the community, this is followed by the moderate-effectiveness strategy, and the low-effectiveness strategy performed the least; furthermore, we found that the moderate-effectiveness strategy is also an effective control strategy.

These simulations clearly shows that MRSA is controllable in a community with IDUs using the control measures, such as the moderate- and high-effectiveness levels of the control strategy described above.

## Discussion

MRSA nasal carriage is shown to be in 33% of the population in the United States [38]. This high number of carriers have the potential to infect individuals they come into physical contact or share intimate materials with [39]. In communities of people in close contact such as inmates and sports teams, the transmission of MRSA is increased due to increased physical contact and increased handling of mutual fomites [40–43]. The dynamics between these communities can be applied to injection drug users who exhibit similar habits as those listed in Table 3.

In today’s IDU communities, the spread and consequences of MRSA infections are of the greatest concern. The resistant bacteria’s severely virulent nature and the associated risk factors of IDU behaviors create an ideal habitat for MRSA. Living in drug houses, sharing needles or saliva are all routine practices for IDUs and all lead to an increase in the risk of MRSA infection [3]. For instance, Cohen et al. [21] found that the use of methamphetamine resulted in an increased number of MRSA skin and soft tissue infections than in those who were not using the drug. MRSA transmission is often times unknown due to asymptomatic colonization [39]. This combined with the increase in risky behaviors contributes to the increase in infected individuals in both IDU subgroups. The known and unique risks IDUs possess contribute to the high infection rate of not only MRSA but well studied HIV transmission [44, 45].

The National Survey on Drug Use and Health (NSDUH) broadens their definition of a risk factor to also include social and mental contributors such as the ideation of a low risk of harm from illicit drug use and availability of the drug [46]. In large metropolitan areas illicit drugs are readily available and according to NSDUH 2015 survey, over 1.5 million people in large metropolitan areas perceived access to illicit drugs as being fairly or very easy versus only 405,000 in non-metropolitan areas [36, 46]. Hence, it is imperative to reduce the risk factor acting on individuals and improve protective factors or decreased likelihood of substance abuse [36, 46]. And these, according to NSDUH, should be the goals of any prevention programs [46].

These are the goals that we aimed to achieve in this study and the results obtained from the control strategies implemented in our model align well with these goals. Furthermore, the results of our model numerical exploration shows that as the risk factor increases, the number of colonized and infected individuals increases as expected. This increase is observed in all the three subgroups under consideration.

In parameterizing our model we have used large metro, large suburb, and rural areas data from the Agency for Healthcare Research and Quality (see Table 2 and Fig. 3). However, we have only used the results for the large meteropolitan areas in our numerical explorations. MRSA is also of concern in suburbs and rural areas due in part to the presence of IDUs [47] and we clearly observed this from the data. Furthermore, the NSDUH 2015 survey found that lifetime use of illicit drugs in large and small metropolitan areas was 113,967 while non-metropolitan areas were 16,644. These statistics bring to light the great need for action in drug abuse interventions in not only metropolitan areas but also suburbs and rural areas. In future work, we will study the impact of movement between large metro, suburb and rural regional areas on the transmission of MRSA among non-IDUs and both low-and high-risk IDUs.

The Agency for Healthcare Research and Quality reported a 21% reduction in hospital acquired conditions (HAC) from 2010-2015 [48], this reduction includes HA-MRSA. During this five-year period, there was a 3 million reduction in the number of cases and $28 billion dollars saved in fighting HACs [48]. Unfortunately, this same results cannot be said of CA-MRSA. To achieve similar significant results as seen with HACs, more research and community efforts will be needed. Nevertheless, these results can be seen with a bright outlook for the future prevention and treatment of MRSA in communities such as IDUs.

## Conclusions

In this section, we summarize some of the main theoretical and epidemiological findings of this study:

- The disease-free equilibrium of the model (1) is locally-asymptotically stable whenever the associated reproduction number (${\mathcal R}_{0}$) is less than unity.
- The model was parametrized by
  - parameterizing the reduced non-IDUs model (5) using AHRQ ICD-6 MRSA regional data for large metro, suburb and rural areas;
  - using risk factors obtained from a constructed ethogram, the non-IDUs parameters are projected onto low- and high-risk IDUs related model parameters.
- The sensitivity analysis of the parameter variations using the associated reproduction number (${\mathcal R}_{N}$) as response function of the reduced model (5) with non-IDUs show the most dominant parameters are the transmission probability (*β*_*N*_) and the recovery rate (*γ*_*N*_).
- The effect of the risk factor was studied, and we found as expected, that as the risk factor increases, the number of colonized and infected increases.
- The study of the vertical downward and upward transitions among non-IDUs and IDUs who are both low-and high-risk users due to change in risky behaviors shows an increase in MRSA cases in the community as individual engage in these risky behaviors. However, with more IDUs entering rehabilitation programs (such as intervention, education, and clean needle exchange programs) MRSA cases reduces.
- The horizontal translation within the subgroups due to disease transmission was also studied by implementing three different strategies: low-effectiveness strategy, moderate-effectiveness strategy, and high-effectiveness strategy. We found that high-effectiveness control strategy is more effective in curtailing MRSA burden in the community; further, we found that the moderate-effectiveness strategy is also an effective control strategy.

## Appendix A: Analysis of the model

### Basic qualitative properties

#### Positivity and boundedness of solutions

For the MRSA transmission model (1) with IDUs to be epidemiologically meaningful, it is important to prove that all its state variables are non-negative for all time. In other words, solutions of the model system (1) with non-negative initial data will remain non-negative for all time *t*>0.

##### **Lemma 1**

Let the initial data *F*(0)≥0, where *F*(*t*)=(*U*_*N*_,*C*_*N*_,*I*_*N*_,*U*_*L*_,*C*_*L*_,*I*_*L*_,*U*_*H*_,*C*_*H*_,*I*_*H*_). Then the solutions *F*(*t*) of the MRSA model *(1)* with IDUs are non-negative for all *t*>0. Furthermore

$$ \limsup_{t \rightarrow \infty} N(t) = \frac{\pi_{H}}{\mu}. $$

where *π*_*H*_=*π*_*N*_+*π*_*L*_+*π*_*H*_ and

$$\begin{array}{@{}rcl@{}} N_{H}(t) &=& U_{N} + C_{N} + I_{N} + U_{L} + C_{L} + I_{L} + U_{H} + C_{H} + I_{H}. \end{array} $$

##### *Proof*

Let *t*_1_=sup{*t*>0:*F*(*t*)>0∈[0,*t*]}. Thus, *t*_1_>0. It follows from the first equation of the system (1), that

$$\begin{aligned} \frac{dU_{N}}{dt} &=\pi_{N} + \alpha_{L} U_{L} + \gamma_{N} I_{N} + \tau C_{N} \\& \quad - \frac{\beta_{N} (C_{N}+I_{N}+C_{L}+I_{L}+C_{H}+I_{H})}{N} \\& \quad - (\omega_{N}+\mu) U_{N} \end{aligned} $$

which can be re-written as

$$\begin{array}{@{}rcl@{}}\frac{d}{dt}\left\{U_{N}(t)~ exp\left[\left(\int^{t_{1}}_{0} \frac{\beta_{N} (C_{N}(\zeta)+I_{N}(\zeta)+C_{L}(\zeta)+I_{L}(\zeta)+C_{H}(\zeta)+I_{H}(\zeta))}{N(\zeta)}d\zeta+ k_{1}t\right)\right] \right\}\\ ~~=~ \pi_{J}~exp\left[\left(\int^{t_{1}}_{0}\frac{\beta_{N} (C_{N}(\zeta)+I_{N}(\zeta)+C_{L}(\zeta)+I_{L}(\zeta)+C_{H}(\zeta)+I_{H}(\zeta))}{N(\zeta)} d\zeta +k_{1}t\right)\right], \end{array} $$

where *k*_1_=*ω*_*N*_+*μ*. Hence,

$$\begin{array}{@{}rcl@{}}U_{N}&(t_{1})& exp\left[\left(\int^{t_{1}}_{0}\frac{\beta_{N} (C_{N}(\zeta)+I_{N}(\zeta)+C_{L}(\zeta)+I_{L}(\zeta)+C_{H}(\zeta)+I_{H}(\zeta))}{N(\zeta)}d\zeta + k_{1}t_{1}\right) \right]-U_{N}(0)\\ &=& \int^{t_{1}}_{0} \pi_{J}~exp\left[\left(\int^{p}_{0}\frac{\beta_{N} (C_{N}(\zeta)+I_{N}(\zeta)+C_{L}(\zeta)+I_{L}(\zeta)+C_{H}(\zeta)+I_{H}(\zeta))}{N(\zeta)}d\zeta + k_{1}p\right)\right]dp \end{array} $$

so that,

$$\begin{array}{@{}rcl@{}} U_{N}(t_{1}) &=&U_{N}(0)~ exp\left[-\left(\int^{t_{1}}_{0}\frac{\beta_{N}(C_{N}(\zeta)+I_{N}(\zeta)+C_{L}(\zeta)+I_{L}(\zeta)+C_{H}(\zeta)+I_{H}(\zeta))}{N(\zeta)}d\zeta + k_{1}t_{1}\right) \right] \\ &\quad+&exp\left[-\left(\int^{t_{1}}_{0}\frac{\beta_{N} (C_{N}(\zeta)+I_{N}(\zeta)+C_{L}(\zeta)+I_{L}(\zeta)+C_{H}(\zeta)+I_{H}(\zeta))}{N(\zeta)}d\zeta + k_{1}t_{1}\right) \right]\\ &\quad\times&\int^{t_{1}}_{0} \pi_{J} ~exp\left[\left(\int^{p}_{0}\frac{\beta_{N} (C_{N}(\zeta)+I_{N}(\zeta)+C_{L}(\zeta)+I_{L}(\zeta)+C_{H}(\zeta)+I_{H}(\zeta))}{N(\zeta)}d\zeta + k_{1}p\right)\right]dp\\ &\quad>&0. \end{array} $$

Similarly, it can be shown that *F*>0 for all *t*>0.

For the second part of the proof, note that 0<*C*_*N*_(*t*)≤*N*(*t*), 0<*I*_*N*_(*t*)≤*N*(*t*), 0<*U*_*L*_(*t*)≤*N*(*t*), 0<*C*_*L*_(*t*)≤*N*(*t*), 0<*I*_*L*_(*t*)≤*N*(*t*), 0<*U*_*H*_(*t*)≤*N*(*t*), 0<*C*_*H*_(*t*)≤*N*(*t*), 0<*I*_*H*_(*t*)≤*N*(*t*).

Adding the human and mosquito component of the MRSA model (1) with IDUs gives

$$\begin{array}{@{}rcl@{}} \frac{dN(t)}{dt} &=& \pi_{H} -\mu N(t), \end{array} $$

where *π*_*H*_=*π*_*N*_+*π*_*L*_+*π*_*H*_.

Hence,

$$\frac{\pi_{H}}{\mu} \leq \liminf_{t\rightarrow\infty}N(t)\leq \limsup_{t\rightarrow\infty}N_{H}(t)=\frac{\pi_{H}}{\mu} $$

as required. □

#### Invariant regions

The MRSA model (1) with IDUs will be analyzed in a biologically-feasible region as follows. Consider the feasible region

$$\Omega \subset \mathbb{R}^{9}_{+}, $$

with,

$$\begin{array}{@{}rcl@{}} \Omega &=& \left\{ U_{N}, C_{N}, I_{N}, U_{L}, C_{L}, I_{L}, U_{H}, C_{H}, I_{H}: \quad N(t)\leq\frac{\pi}{\mu}\right\}. \end{array} $$

##### **Lemma 2**

The region $\Omega \subset \mathbb {R}^{9}_{+}$ is positively-invariant for the MRSA model *(1)* with IDUs with non-negative initial conditions in $\mathbb {R}^{9}_{+}$.

##### *Proof*

It follows from summing equations of model (1) that

$$\begin{array}{@{}rcl@{}}  \frac{dN(t)}{dt} &=& \Pi -\mu N - \delta_{N} I_{N} - \delta_{L} I_{L} - \delta_{H} I_{H} \\ &\leq& \Pi -\mu N  \end{array} $$

Hence, $\frac {dN(t)}{dt}\leq 0$, if $N(0)\geq \frac {\Pi }{\mu } $. Thus, $N(t)\leq N(0)e^{-\mu t}+\frac {\Pi }{\mu }\left (1-e^{-\mu t}\right)$. In particular, $N(t)\leq \frac {\Pi }{\mu }$.

Thus, the region *Ω* is positively-invariant. Furthermore, if $N(0)>\frac {\Pi }{\mu }$, then either the solutions enters *Ω* in finite time, or *N*(*t*) approaches $\frac {\Pi }{\mu }$ asymptotically. Hence, the region *Ω* attracts all solutions in $ \mathbb {R}^{9}_{+}$. □

## Appendix B: Stability of disease-free equilibrium (DFE) and the basic reproduction number $\mathcal {R}_{0}$

The conditions for stability of the equilibria of the model (1) are stated in this section.

The MRSA model (1) with IDUs has a disease-free equilibrium (DFE), obtained by setting the right-hand sides of the equations in the model to zero, given by

10 $$\begin{array}{@{}rcl@{}}  \mathcal{E}_{0} &=& \left(U^{*}_{N}, C^{*}_{N}, I^{*}_{N}, U^{*}_{L}, C^{*}_{L}, I^{*}_{L}, U^{*}_{H}, C^{*}_{H}, I^{*}_{H}\right)~ \\ &=&\left(U^{*}_{N},0,0,U^{*}_{L},0,0,U^{*}_{H},0,0\right). \end{array} $$

where

$$\begin{array}{@{}rcl@{}} U^{*}_{N} &=& \frac{\pi_{N}\left(g_{2}g_{7}-\alpha_{H}\omega_{L}\right)+\alpha_{L}\pi_{L}g_{3}+\alpha_{L}\alpha_{H}\pi_{H}}{g_{1}g_{2}g_{3}-\alpha_{H}\omega_{L}g_{1}-\omega_{N}\alpha_{L}g_{3}},\\ U^{*}_{L} &=& \frac{\pi_{N}\omega_{N}g_{3}+\pi_{L}g_{1}g_{3}+\alpha_{H}\pi_{H}g_{1}}{g_{1}g_{2}g_{3}-\alpha_{H}\omega_{L}g_{1}-\omega_{N}\alpha_{L}g_{3}},\\ U^{*}_{H} &=& \frac{\omega_{L}\omega_{N}\pi_{N}+\omega_{L}\pi_{L}g_{1}+\pi_{H}\left(g_{1}g_{2}-\omega_{N}\alpha_{L}\right)}{g_{1}g_{2}g_{3}-\alpha_{H}\omega_{L}g_{1}-\omega_{N}\alpha_{L}g_{3}}, \end{array} $$

with *g*_1_=*ω*_*N*_+*μ*, *g*_2_=*ω*_*L*_+*α*_*L*_+*μ*, *g*_3_=*α*_*H*_+*μ*.

The linear stability of $\mathcal {E}_{0}$ can be established using the next generation operator method on the system (1). Taking, *C*_*N*_,*I*_*N*_,*C*_*L*_,*I*_*L*_,*C*_*H*_,*I*_*H*_, as the infected compartments, then using the notation in [19], the Jacobian matrices *F* and *V* for the new infection terms and the remaining transfer terms are respectively given by,

$$ {F = \left(\begin{array}{cccccc} \frac{\beta_{N} U^{*}_{N}}{N^{*}} & \frac{\beta_{N} U^{*}_{N}}{N^{*}} & \frac{\beta_{N} U^{*}_{N}}{N^{*}} & \frac{\beta_{N} U^{*}_{N}}{N^{*}} & \frac{\beta_{N} U^{*}_{N}}{N^{*}} & \frac{\beta_{N} U^{*}_{N}}{N^{*}} \\ 0 & 0 & 0 & 0 & 0 & 0 \\ \frac{\beta_{L} U^{*}_{L}}{N^{*}} & \frac{\beta_{L} U^{*}_{L}}{N^{*}} & \frac{\beta_{L} U^{*}_{L}}{N^{*}} & \frac{\beta_{L} U^{*}_{L}}{N^{*}} & \frac{\beta_{L} U^{*}_{L}}{N^{*}} & \frac{\beta_{L} U^{*}_{L}}{N^{*}} \\ 0 & 0 & 0 & 0 & 0 & 0 \\ \frac{\beta_{H} U^{*}_{H}}{N^{*}} & \frac{\beta_{H} U^{*}_{H}}{N^{*}} & \frac{\beta_{H} U^{*}_{H}}{N^{*}} & \frac{\beta_{H} U^{*}_{H}}{N^{*}} & \frac{\beta_{H} U^{*}_{H}}{N^{*}} & \frac{\beta_{H} U^{*}_{H}}{N^{*}} \\ 0 & 0 & 0 & 0 & 0 & 0 \\ \\ \end{array}\right)} $$

and

11 $$ {V = \left(\begin{array}{cccccc} k_{2} & 0 & -\alpha_{L} & 0 & 0 & 0\\ -\sigma_{N} & k_{3} & 0 & -\alpha_{L} & 0 & 0\\ -\omega_{N} & 0 & k_{5} & 0 & -\alpha_{H} & 0\\ 0 & -\omega_{N} & -\sigma_{L} & k_{6} & 0 & -\alpha_{H}\\ 0 & 0 & -\omega_{L} & 0 & k_{7} & 0\\ 0 & 0 & 0 & -\omega_{L} & -\sigma_{H} & k_{8}\\ \end{array}\right)}  $$

where *k*_1_ = *ω*_*N*_+*μ*, *k*_2_ = *τ*_*N*_+*σ*_*N*_+*ω*_*N*_+*μ*, *k*_3_ = *γ*_*N*_+*ω*_*N*_+*μ*+*δ*_*N*_, *k*_4_ = *ω*_*L*_+*α*_*L*_+*μ*, *k*_5_ = *τ*_*L*_+*ω*_*L*_+*α*_*L*_+*σ*_*L*_+*μ*, *k*_7_ = *α*_*H*_+*μ*, *k*_6_ = *ω*_*L*_+*α*_*L*_+*γ*_*L*_+*μ*+*δ*_*L*_, *k*_8_ = *τ*_*H*_+*α*_*H*_+*σ*_*H*_+*μ*, *k*_9_ = *α*_*H*_+*γ*_*H*_+*μ*+*δ*_*H*_.

It follows that the basic reproduction number of the MRSA model (1) with IDUs, is given by:

12 $$\begin{array}{@{}rcl@{}} \mathcal{R}_{0} &=& \rho\left(FV^{-1}\right) = \mathcal{R}_{N} + \mathcal{R}_{L} + \mathcal{R}_{H},  \end{array} $$

where *ρ* is the spectral radius and

$$\begin{array}{@{}rcl@{}} \mathcal{R}_{N} &=& \beta_{N}U^{*}_{N}\left[k_{7}\omega_{N}(k_{3}k_{8}+\omega_{L}k_{3}+k_{8}\alpha_{L})\sigma_{L}\right. \\[-1pt] &&+(k_{6}k_{8}+\omega_{N}k_{8}+\omega_{N}\omega_{L}-\omega_{L}\alpha_{H})(k_{5}k_{7}-\omega_{L}\alpha_{H})\sigma_{N} \\[-1pt] &&+\omega_{N}\omega_{L}(k_{3}k_{6}+k_{3}\alpha_{H}+\alpha_{H}\alpha_{L}-\omega_{N}\alpha_{L})\sigma_{H} \\[-1pt] &&\left.+(k_{5}k_{7}+k_{7}\omega_{N}+\omega_{L}\omega_{N}-\omega_{L}\alpha_{H})\right.\\&& \left. \times(k_{3}k_{6}k_{8}-k_{3}\omega_{L}\alpha_{H}-k_{8}\omega_{N}\alpha_{L})\right] \\[-1pt] &&/N^{*}(k_{3}k_{6}k_{8}-k_{3}\omega_{L}\alpha_{H}-k_{8}\omega_{N}\alpha_{L})\\&&\times(k_{2}k_{5}k_{7}-k_{2}\omega_{L}\alpha_{H}-k_{7}\omega_{N}\alpha_{L})\\ \mathcal{R}_{L} &=& \beta_{L}U^{*}_{L}\left[k_{2}k_{7}(k_{3}k_{8}+\omega_{L}k_{3}+\alpha_{L}k_{8})\sigma_{L} \right.\\[-1pt] &&+k_{7}\alpha_{L}(k_{6}k_{8}+k_{8}\omega_{N}+\omega_{N}\omega_{L}-\omega_{L}\alpha_{H})\sigma_{N} \\[-1pt] &&+k_{2}\omega_{L}(k_{3}k_{6}+k_{3}\alpha_{H}+\alpha_{L}\alpha_{H}-\omega_{N}\alpha_{L})\sigma_{H} \\[-1pt] &&\left.+(k_{2}k_{7}+k_{2}\omega_{L}+k_{7}\alpha_{L})(k_{3}k_{6}k_{8}-k_{3}\omega_{L}\alpha_{H}\right.\\[-1pt] &&\left.-k_{8}\omega_{N}\alpha_{L})\right] /N^{*}(k_{3}k_{6}k_{8}-k_{3}\omega_{L}\alpha_{H}-k_{8}\omega_{N}\alpha_{L})\\[-1pt] &&\times(k_{2}k_{5}k_{7}-k_{2}\omega_{L}\alpha_{H}-k_{7}\omega_{N}\alpha_{L})\\ \mathcal{R}_{H} &=& \beta_{H}U^{*}_{H}\left[\alpha_{L}\alpha_{H}(k_{6}k_{8}+k_{8}\omega_{N}+\omega_{N}\omega_{L}-\omega_{L}\alpha_{H})\sigma_{N} \right.\\[-1pt] &&+k_{2}\alpha_{H}(k_{3}k_{8}+k_{3}\omega_{L}+k_{8}\alpha_{L})\sigma_{L} \\[-1pt] &&+(k_{3}k_{6}+k_{3}\alpha_{H}+\alpha_{L}\alpha_{H}-\omega_{N}\alpha_{L})(k_{2}k_{5}-\omega_{N}\alpha_{L})\sigma_{H} \\[-1pt] &&\left.+(k_{2}k_{5}+k_{2}\alpha_{H}+\alpha_{L}\alpha_{H}-\omega_{N}\alpha_{L})(k_{3}k_{8}k_{6}-k_{8}\omega_{N}\alpha_{L}\right. \\[-1pt] &&\left.-k_{3}\omega_{L}\alpha_{H})\right]/N^{*}(k_{3}k_{6}k_{8}-k_{3}\omega_{L}\alpha_{H}-k_{8}\omega_{N}\alpha_{L}) \\[-1pt] &&\times(k_{2}k_{5}k_{7}-k_{2}\omega_{L}\alpha_{H}-k_{7}\omega_{N}\alpha_{L}). \end{array} $$

Furthermore, the expression $\mathcal {R}_{N}$ is the number of secondary infections among the non-injection drug users, $\mathcal {R}_{L}$ is the number of secondary infections among the low-risk injection drug users, $\mathcal {R}_{H}$ is the number of secondary infections among the high-risk injection drug users. These expressions ($\mathcal {R}_{N}, \mathcal {R}_{L}, \mathcal {R}_{H}$) further show the secondary infection in each sub-groups due to both horizontal and vertical transition of infectious individuals. Hence, using Theorem 2 in [19], the following result is established.

### **Lemma 3**

The disease-free equilibrium (${\mathcal E}_{0}$) of the MRSA model *(1)* with IDUs is locally asymptotically stable (LAS) if $\mathcal {R}_{0} < 1$ and unstable if $\mathcal {R}_{0} >1$.

The basic reproduction number $\mathcal {R}_{0}$ is defined as the average number of new infections that result from one infectious individual in a population that is fully susceptible [16–19]. The epidemiological significance of Lemma 3 is that MRSA will be eliminated from the community if the reproduction number (${{\mathcal R}}_{0}$) can be brought to (and maintained at) a value less than unity.

### The basic reproduction number when *ω*_*N*_=*ω*_*L*_=*α*_*L*_=*α*_*H*_=0

Suppose the vertical upward and downward transition between the subgroups are absent, that is, *ω*_*N*_=0, *ω*_*L*_=0, *α*_*L*_=0, *α*_*H*_=0, then the DFE (10) becomes

$$\begin{array}{@{}rcl@{}} \mathcal{E}_{0} &=& (U^{*}_{N}, C^{*}_{N}, I^{*}_{N}, U^{*}_{L}, C^{*}_{L}, I^{*}_{L}, U^{*}_{H}, C^{*}_{H}, I^{*}_{H})\\ &=&\left(\frac{\pi_{N}}{\mu},0,0,\frac{\pi_{L}}{\mu},0,0,\frac{\pi_{H}}{\mu},0,0\right). \end{array} $$

and the V matrix (11) is given as

$$ V = \left(\begin{array}{cccccc} k_{1} & 0 & 0 & 0 & 0 & 0 \\ -\sigma_{N} & k_{2} & 0 & 0 & 0 & 0 \\ 0 & 0 & k_{3} & 0 & 0 & 0 \\ 0 & 0 & -\sigma_{L} & k_{4} & 0 & 0 \\ 0 & 0 & 0 & 0 & k_{5} & 0 \\ 0 & 0 & 0 & 0 & -\sigma_{H} & k_{6} \\ \end{array}\right) $$

The basic reproduction number of the MRSA model (1) with IDUs, in this case is given by:

13 $$\begin{array}{@{}rcl@{}} \mathcal{R}_{0} &=& \rho\left(FV^{-1}\right) ~~=~~ \mathcal{R}_{N} + \mathcal{R}_{L} + \mathcal{R}_{H}, \end{array} $$

where

$$\begin{array}{@{}rcl@{}} \mathcal{R}_{N}&=&\frac{\beta_{N} U^{*}_{N}(k_{3}+\sigma_{N})}{k_{2}k_{3}N^{*}},\quad \mathcal{R}_{L}~=~\frac{\beta_{L} U^{*}_{L}(k_{6}+\sigma_{L})}{k_{5}k_{6}N^{*}},\\ \mathcal{R}_{H}&=& \frac{\beta_{H} U^{*}_{H}(k_{8}+\sigma_{H})}{k_{7}k_{8}N^{*}}. \end{array} $$

It should be noted that the reproduction number stated in Eq. (12) gives the reproduction number in Eq. (13) in the absence of vertical downward and upward transition, that is if *ω*_*N*_=*ω*_*L*_=*α*_*L*_=*α*_*H*_=0.

## Abbreviations

AHRQ: Agency for Healthcare and research and quality
CA-MRSA: Community-acquired MRSA
DAP: Drug abuse program
HAC: Hospital-acquired conditions
HA-MRSA: Hospital-acquired MRSA
ICD: International classification of diseases
IDUs: Injection drug users
MRSA: Methicillin-resistant Staphylococcus aureus
NSDUH: National survey on drug use and health
